# Synthesis of a novel Cu/DPA-MOF/OP/CS hydrogel with high capability in antimicrobial studies

**DOI:** 10.3389/fchem.2023.1236580

**Published:** 2023-08-11

**Authors:** Hanadi Hadi Jasim Al-Khafaji, Ali Alsalamy, Mohammed Abed Jawad, Hind Ali Nasser, Ashour H. Dawood, Saif Yaseen Hasan, Irfan Ahmad, M. Abdulfadhil Gatea, Waleed Khaled Younis Albahadly

**Affiliations:** ^1^ College of Dentistry, Al-Mustaqbal University, Babylon, Iraq; ^2^ College of Technical Engineering, Imam Ja’afar Al-Sadiq University, Al-Muthanna, Iraq; ^3^ Department of Medical Laboratories Technology, Al-Nisour University College, Al-Mansour, Iraq; ^4^ College of Pharmacy, Al-Ayen University, Nasiriyah, Thi-Qar, Iraq; ^5^ Department of Medical Engineering, Al-Esraa University College, Baghdad, Iraq; ^6^ College of Health and Medical Technology, National University of Science and Technology, Nasiriyah, Thi-Qar, Iraq; ^7^ Department of Clinical Laboratory Sciences, College of Applied Medical Sciences, King Khalid University, Abha, Saudi Arabia; ^8^ Technical Engineering Department College of Technical Engineering, The Islamic University, Najaf, Iraq; ^9^ Department of Physics, College of Science, University of Kufa, Kufa, Iraq; ^10^ Medical Technical College, College of Pharmacy, University of Al-Ameed, Karbala, Iraq

**Keywords:** hydrogel, oxidized pectin, chitosan, copper metal–organic framework, antimicrobial study

## Abstract

Today, with the indiscriminate use of antibiotics, we face the resistance of some bacterial strains against some antibiotics. Therefore, it is essential to report and synthesize new compounds with antimicrobial properties. A novel copper/dipicolinic acid–metal–organic framework cross-linked oxidized pectin and chitosan (Cu/DPA-MOF/OP/CS) hydrogel polymer was synthesized under environmental conditions with the controllable process, which uses biodegradable polymer compounds such as pectin and chitosan in its structure. The efficient physicochemical features of the synthesized Cu/DPA-MOF/OP/CS hydrogel using SEM, FT-IR, TGA, BET, XRD, and EDS/mapping were identified and confirmed. The newly synthesized Cu/DPA-MOF/OP/CS hydrogel showed activity against Gram-positive and Gram-negative bacterial strains and fungal species, and significant antibacterial and antifungal properties were observed. In antibacterial activity, the MIC against Gram-positive species was in the range of 16–128 mg/mL, the MIC against Gram-negative species was in the range of 64–256 mg/mL, and the MIC against fungal species was in the range of 128–512 mg/mL. In antimicrobial evaluations, in addition to the MIC test, the MBC test, the MFC test, and the IZD test were performed, and the results were reported. The results were compared with commercial antibiotics in the market. Development of novel nanostructures based on hydrogel polymers with distinctive functionality can affect the performance of these nanostructures in different areas.

## 1 Introduction

In recent years, the widespread and generally unnecessary use of drugs has caused the resistance of a wide range of pathogenic microbes. This has resulted in threats and health concerns, increased treatment costs, and patient deaths (Otter et al., 2015). Providing new compounds with antimicrobial properties is one of the goals suggested to prevent these cases. As is known, nanotechnology is developing, and nanostructures can have potential activities in this field. Various nanocompounds with biological properties such as anticancer (Wani et al., 2016), anticoagulant (Zaky et al., 2023), antiviral (Balagna et al., 2021), and antimicrobial activities (Al-Jumaili et al., 2017) have been reported. The use of metals causes significant biological properties in these compounds (Khan et al., 2018; Zare et al., 2019). Copper is known as one of the transition metals of the periodic table with biological properties. So far, new complex and nanocompounds containing copper with biological properties such as antibacterial and antifungal activities have been reported (de Araújo et al., 2017; Marković et al., 2018; Dimitrijević et al., 2020; Li et al., 2023). Recently, there have been reports on the use of this metal in advanced nanocompounds such as metal–organic frameworks (MOFs) with biological properties (Jo et al., 2019; Sun et al., 2021; Lin et al., 2023). The unique properties of MOF compounds have attracted the attention of biomedical aspects (Al-Rowaili et al., 2018; Giliopoulos et al., 2020). Excellent physical and chemical properties, such as high porosity, high thermal stability, and high reactivity, are the most critical features of these compounds (Razavi and Morsali, 2019; Ghanbari et al., 2020). These properties and characteristics have caused the use of these compounds to be reported as potent biomedical agents (Babucci et al., 2020; Ahmadi et al., 2021; Li et al., 2021; Han et al., 2022). Although these compounds have significant physicochemical properties, according to their applications, biocompatibility and biodegradability need to be improved.

These compounds can connect with organic polymers and create novel polymer compounds (Han et al., 2022). Today, the use of polymers in life is inevitable. Polymers have many applications, such as in the building and automotive industries, binder materials, cables and pipes, and membrane materials (Sun, 2019; Nurazzi et al., 2021; Hu et al., 2022). Polymers are also found in abundance in nature, such as DNA, cellulose, starch, pectin, and chitosan. Polymer compounds with significant properties can be produced using natural polymer compounds. For example, recently, there have been reports on the use of pectin and other compounds, such as metal–organic frameworks and hybrid materials, in synthesizing new polymers with biomedical and drug delivery applications (Rascón-Chu et al., 2019; Li et al., 2021; Kiadeh et al., 2021). Chitosan is mentioned among other natural polymers. Chitosan with numerous nanocompounds can create novel polymer compounds with high biological capabilities. Among others, we can refer to the reports of chitosan–ZnO nanoparticles, chitosan-functionalized MoS_2_ hybrids, halloysite nanotubes by chitosan grafting, etc., with anticancer and antimicrobial properties (Ghaffari et al., 2020; Kasinathan et al., 2020).

Therefore, it can be expected that a metal–organic framework containing copper and natural polymers such as chitosan and pectin can synthesize new nanostructures with its unique biological properties.

Microwave is one of the most efficient procedures for the synthesis of different materials with diverse applications (Sargazi et al., 2018). This method is not only fast, affordable, and controllable, but its operational process is also environmentally friendly. These properties have distinguished the microwave method from other conventional methods (Zheng et al., 2022).

In this study, we investigated that whether the polymer synthesized by the hydrogel method using the aforementioned materials after identifying and confirming the structure was subjected to antimicrobial evaluations such as antifungal and antibacterial evaluations.

## 2 Materials and methods

### 2.1 Materials

All materials such as copper(II) nitrate trihydrate, 2, 6-pyridine dicarboxylic acid, chitosan (10 mg/mL acetic acid: water), pectin (pectin from citrus peel, impurities ≤10% moisture), and solvents with high purity were obtained from Merck and Sigma-Aldrich. The cultures such as Mueller Hinton agar and Mueller Hinton broth were obtained from Sigma-Aldrich. The bacterial and fungal strains were obtained from American Type Culture Collection.

### 2.2 Synthesis of copper/dipicolinic acid–metal–organic framework cross-linked oxidized pectin and chitosan hydrogel

For the synthesis of Cu/DPA-MOF/OP/CS hydrogel, the first copper/dipicolinic acid-metal–organic framework (Cu/DPA-MOF) was synthesized as follows: in 25 mL bidet water (double distilled water), copper (II) nitrate trihydrate, as a source of metal (0.1 mmol), and dipicolinic acid, as a linker (0.1 mmol), were placed under microwave irradiation at ambient temperature for 25 min with a microwave power of 320 W. Then, the synthesized Ti/DPA-MOF, used for the next step, was isolated by nanofiltration, washed three times with bidet water and EtOH, and dried for 48 h under vacuum at ambient temperature.

In the next step, under stirring at 30°C, oxidized pectin (2 g) was dissolved in bidet water (10 mL). In another receptacle, chitosan (2 g) was dissolved in bidet water (10 mL) under the aforementioned conditions (Salama and Aziz, 2020). The synthesized Cu/DPA-MOF **(**300 mg) was added to the oxidized pectin solution and stirred (800 rpm) for 1 h at 30°C. The chitosan solution was added drop by drop to the Cu/DPA-MOF/oxidized pectin (Cu/DPA-MOF/OP) solution at 30°C under stirring for 1 h. Finally, the container containing the admixture was placed in water at 37°C for 4 h.

### 2.3 Antimicrobial studies of Cu/DPA-MOF and Cu/DPA-MOF/OP/CS hydrogel

*In vitro* minimum inhibitory/fungicidal/bactericidal concentrations and the disk inhibition zone diameter (DIZD) for synthesized Cu/DPA-MOF and Cu/DPA-MOF/OP/CS hydrogel were tested. In antimicrobial studies, standard guidelines reported by previous work were used (Hosseinzadegan et al., 2020; Zeraati et al., 2022). The minimum inhibitory concentration/fungicidal concentration (MIC and MFC) for antifungal studies and MIC and minimum bactericidal concentration (MBC) for antibacterial studies were reported.

#### 2.3.1 *In vitro* minimum inhibitory concentration (MIC)method

For the *in vitro* minimum inhibitory test, concentrations of 1, 2, 4, 8,16, 32, 64, 128, 256, 512, 1024, and 2048 μg/mL of Cu/DPA-MOF and Cu/DPA-MOF/OP/CS hydrogel were dispersed in bidet water. A volume of 100 μL of the prepared concentrations was transferred to the microplate wells. Then, 100 μL of the liquid culture medium (for antibacterial investigation, Mueller Hinton broth, and for antifungal investigation, Dextrose Tryptone broth) was added. Finally, 10 μL of the bacterial/fungal suspension with the prepared concentration of 1 × 10^5^ CFU/mL (colony-forming unit/mL) was added to the wells. The wells were incubated for a suitable period (48 h) at an appropriate temperature (37°C to check antibacterial activity and 27°C to check antifungal activity). A lower concentration that was clear was reported as MBC/MFC. It is noted that the last row of each plate was only a mixture of the culture medium and bacterial/fungal suspension as a control without derivatives (Hosseinzadegan et al., 2020; Zeraati et al., 2022). The results were averaged after three repetitions.

#### 2.3.2 *In vitro* minimum fungicidal/bactericidal concentration (MFC/MBC) method

For the *in vitro* minimum fungicidal/bactericidal concentration test, the MIC and five diluted concentrations of the previous step were cultured on the appropriate agar culture medium (for antibacterial activity, Mueller Hinton agar, and for antifungal activity, Dextrose Tryptone agar). Then, they were incubated for a suitable period (72 h) at an appropriate temperature (37°C to check antibacterial activity and 27°C to check antifungal activity). The concentration at which bacteria/fungi did not grow was reported as the minimum fungicidal/bactericidal concentration. The results were averaged after three repetitions (Hosseinzadegan et al., 2020; Zeraati et al., 2022).

#### 2.3.3 *In vitro* disk inhibition zone diameter (DIZD) method

For determining the *in vitro* disk inhibition zone diameter, first, bacterial/fungal species were cultured on a suitable agar culture medium (for antibacterial activity, Mueller Hinton agar and for antifungal activity, Dextrose Tryptone agar), and a disk blank was placed on it. Then, the minimum inhibitory concentrations of Cu/DPA-MOF and Cu/DPA-MOF/OP/CS hydrogel were dispersed in bidet water. A measure of 10 μL of the prepared concentration was injected into a disk blank. The plates were incubated for a suitable time (48 h) at an appropriate temperature (37°C to check antibacterial activity and 27°C to check antifungal activity). Finally, the diameter of the created halo was measured using a caliper. The results were averaged after three repetitions (Zeraati et al., 2022).

## 3 Results and discussion

### 3.1 Characterization and structure prediction of the copper/dipicolinic acid–metal–organic framework cross-linked oxidized pectin and chitosan (Cu/DPA-MOF/OP/CS) hydrogel

In two steps, the novel Cu/DPA-MOF/OP/CS hydrogel was synthesized. In the first step, Cu/DPA-MOF was synthesized using copper(II) nitrate trihydrate (0.1 mmol) and dipicolinic acid under microwave irradiation. In the second step, the Cu/DPA-MOF/OP/CS hydrogel was synthesized using Cu/DPA-MOF, oxidized pectin, and chitosan.

To characterize, confirm, and predict the structure of the Cu/DPA-MOF/OP/CS hydrogel, techniques such as N_2_ adsorption/desorption isotherm, FT-IR spectrum, XRD patterns, SEM images, thermal stability curve, EDS elemental analysis, and mapping graph were used.

**FIGURE 1:**
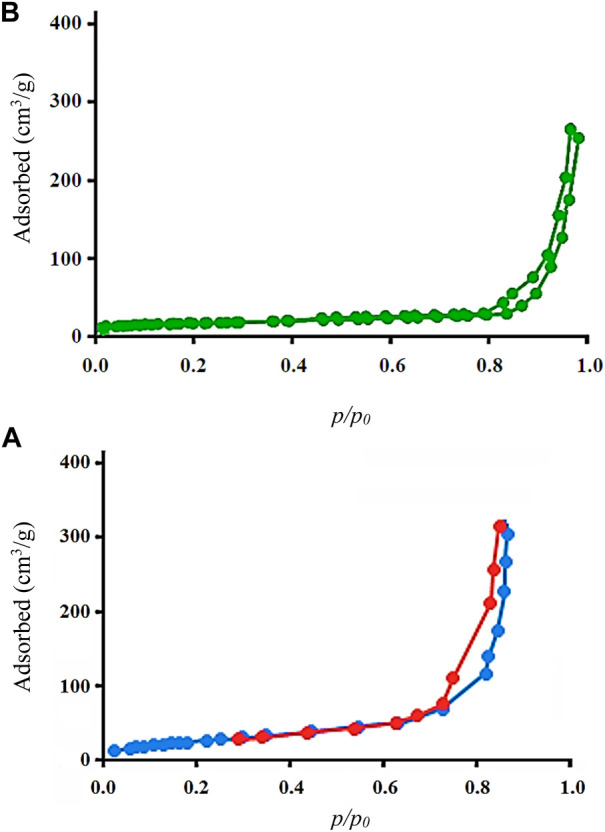
N_2_ adsorption/desorption isotherms of Cu/DPA-MOF **(A)** and Cu/DPA-MOF/OP/CS hydrogel **(B)**.

The curves in Figure 1 show the N_2_ adsorption/desorption isotherm related to Cu/DPA-MOF (A) and Cu/DPA-MOF/OP/CS hydrogel (B). Based on the BET results, the specific surface area for Cu/DPA-MOF and Cu/DPA-MOF/OP/CS hydrogel was obtained as 28.200 m^2^/g and 37.700 m^2^/g, respectively. Based on previous studies, the high specific surface area is an essential factor in the reactivity, properties, and performance of nanoparticles (Asiri et al., 2022; Zeraati et al., 2022). Therefore, it can be suggested that the polymerization of Cu/DPA-MOF Cu/DPA by oxidized pectin and chitosan caused a significant increase in the specific surface area.

**FIGURE 2:**
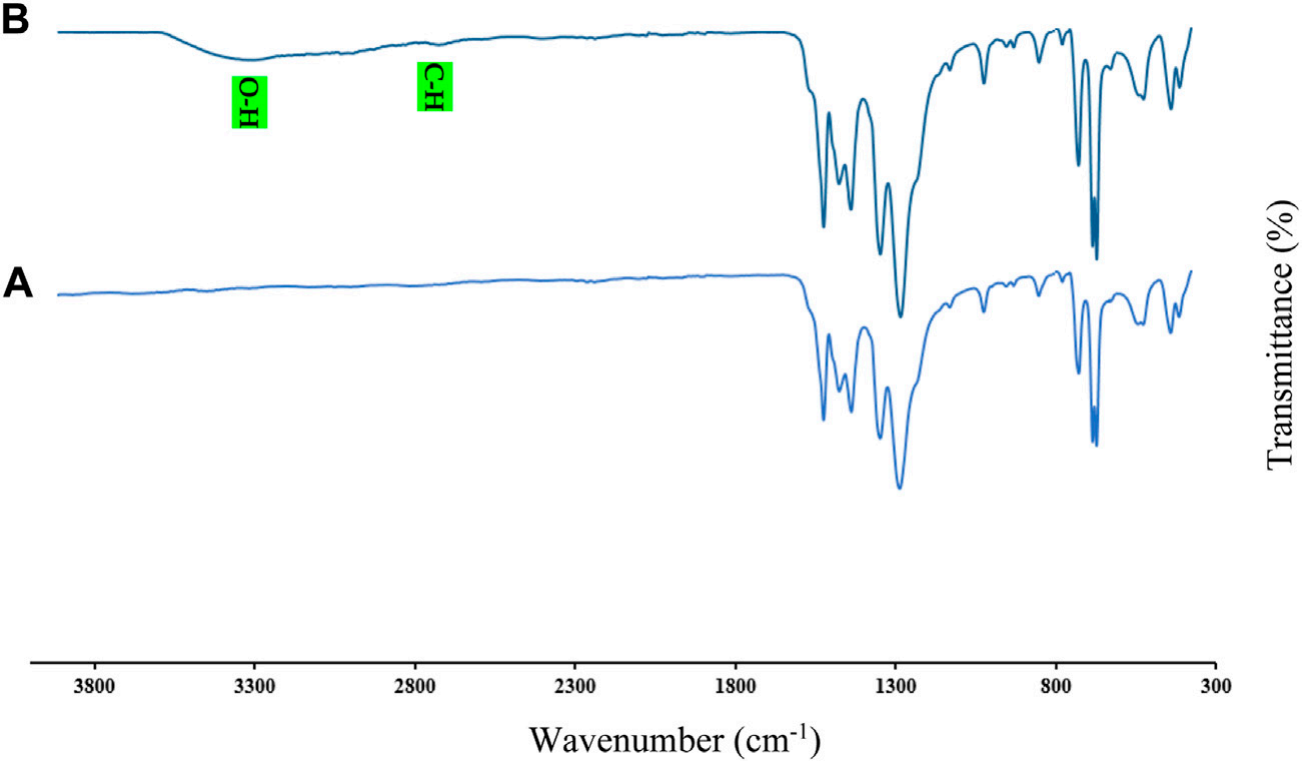
FT-IR spectra of Cu/DPA-MOF **(A)** and Cu/DPA-MOF/OP/CS hydrogel **(B)**.

As is known, one of the most important analyses to identify and confirm the structure of organic compounds and polymers is the FT-IR spectrum. Absorptions related to functional groups and bonds between atoms can be recognized and confirmed using the FT-IR spectrum. The FT-IR spectra of Cu/DPA-MOF (A) and the Cu/DPA-MOF/OP/CS hydrogel (B) were prepared after the synthesis and are shown in Figure 2. In the FT-IR spectra of Cu/DPA-MOF and Cu/DPA-MOF/OP/CS hydrogel, significant absorptions such as Cu–O, C–O, C=C, C=N, and C=O were observed in regions 400–600 cm^-1^ (Elango et al., 2018), 1000–1100 cm^-1^, 1300–1400 cm^-1^, 1450 cm^-1^, and 1600 cm^-1^, respectively. In the FT-IR spectrum of Cu/DPA-MOF/OP/CS hydrogel, absorption related to C–H of SP^3^ carbons below 3000 cm^-1^ and O–H groups in the region of 3300 cm^-1^ were observed (Bakhshi et al., 2022; Jasim et al., 2022).

**FIGURE 3:**
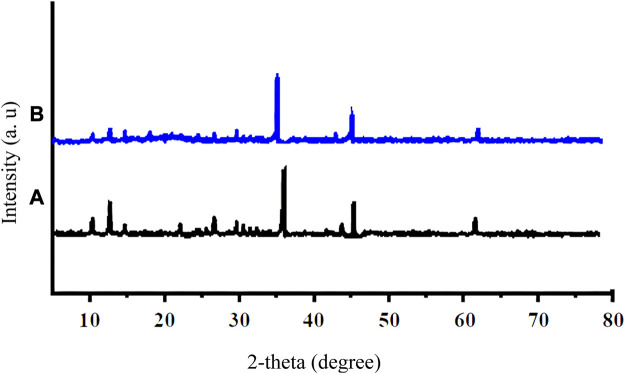
XRD patterns of Cu/DPA-MOF **(A)** and Cu/DPA-MOF/OP/CS hydrogel **(B)**.

By XRD patterns presented in Figure 3, the size of Cu/DPA-MOF (Figure 3A) and Cu/DPA-MOF/OP/CS hydrogel (Figure 3B) was calculated using Scherrer’s method, according to the relevant equation and previous reports (Saqezi et al., 2022), and was found to be 61 and 78 nm, respectively. In XRD patterns, the planes [111], [200], and [220] related to copper nanoparticles were observed at 36°, 45°, and 62°, respectively (Gan et al., 2015; Elango et al., 2018).

**FIGURE 4:**
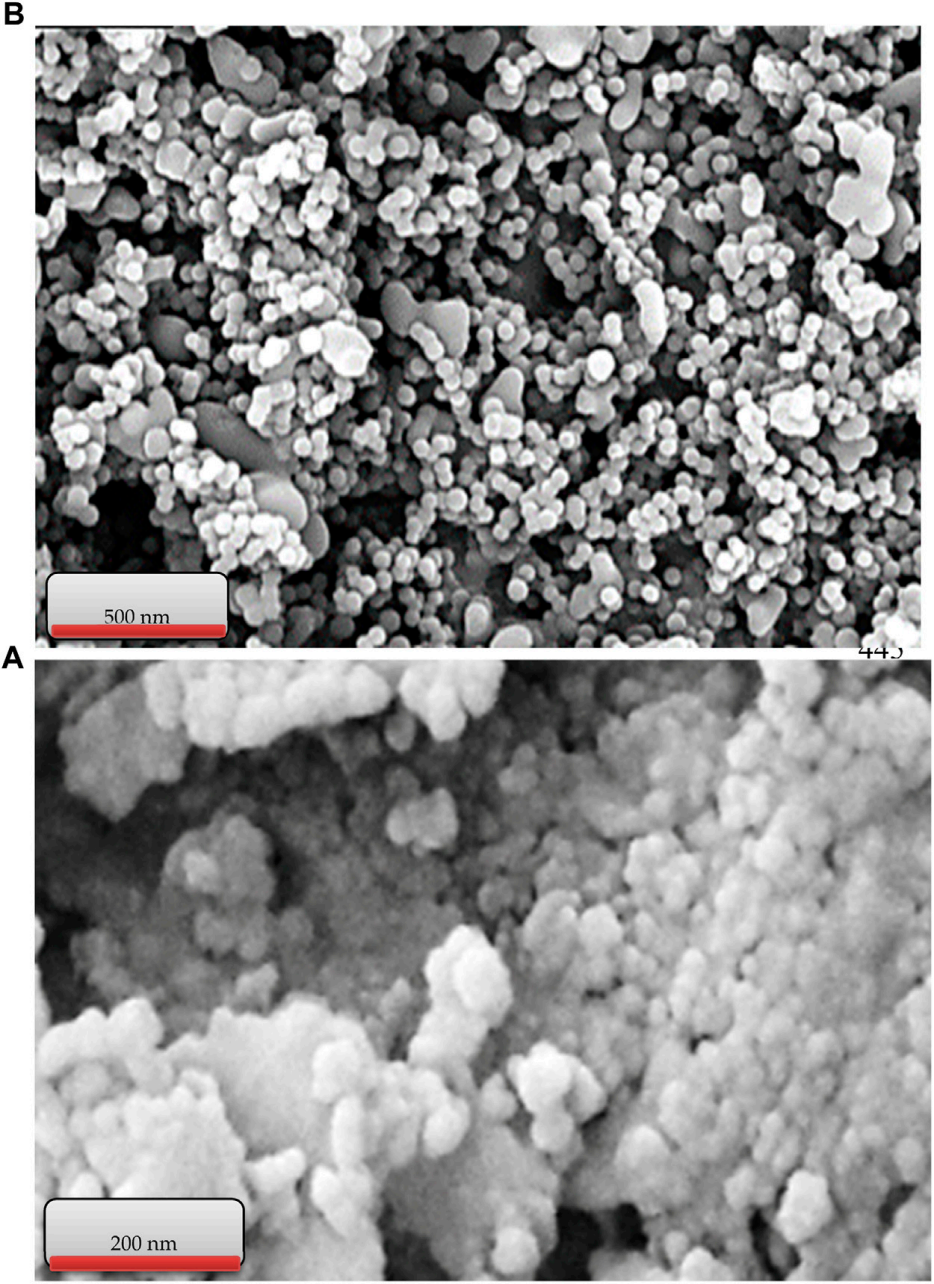
SEM images of Cu/DPA-MOF **(A)** and Cu/DPA-MOF/OP/CS hydrogel **(B)**.

Figure 4 shows the SEM images of Cu/DPA-MOF (A) and Cu/DPA-MOF/OP/CS hydrogel (B). The nano-sized synthesized compounds and the same morphology of the nanostructures are observed in the figure. Therefore, in addition to XRD patterns, SEM images confirm the nano-sized structure of synthetic compounds.

**FIGURE 5:**
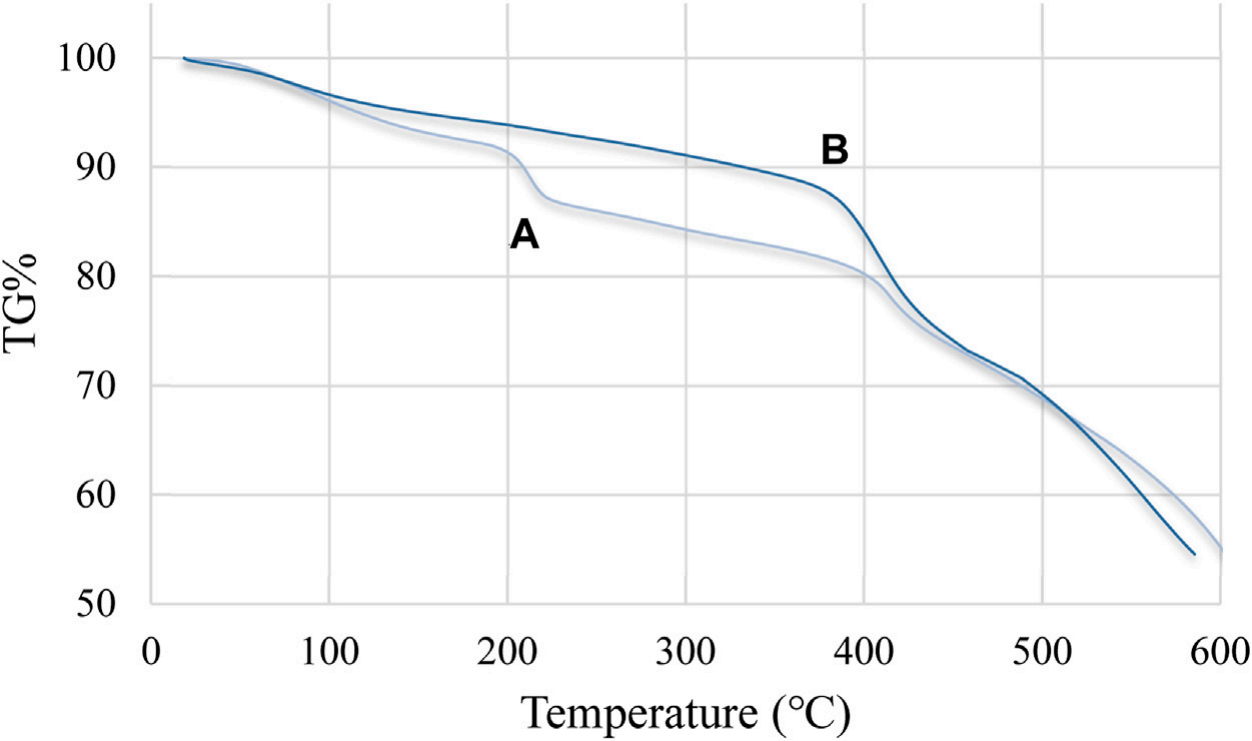
Thermal behavior of Cu/DPA-MOF **(A)** and Cu/DPA-MOF/OP/CS hydrogel **(B)**.

**FIGURE 6:**
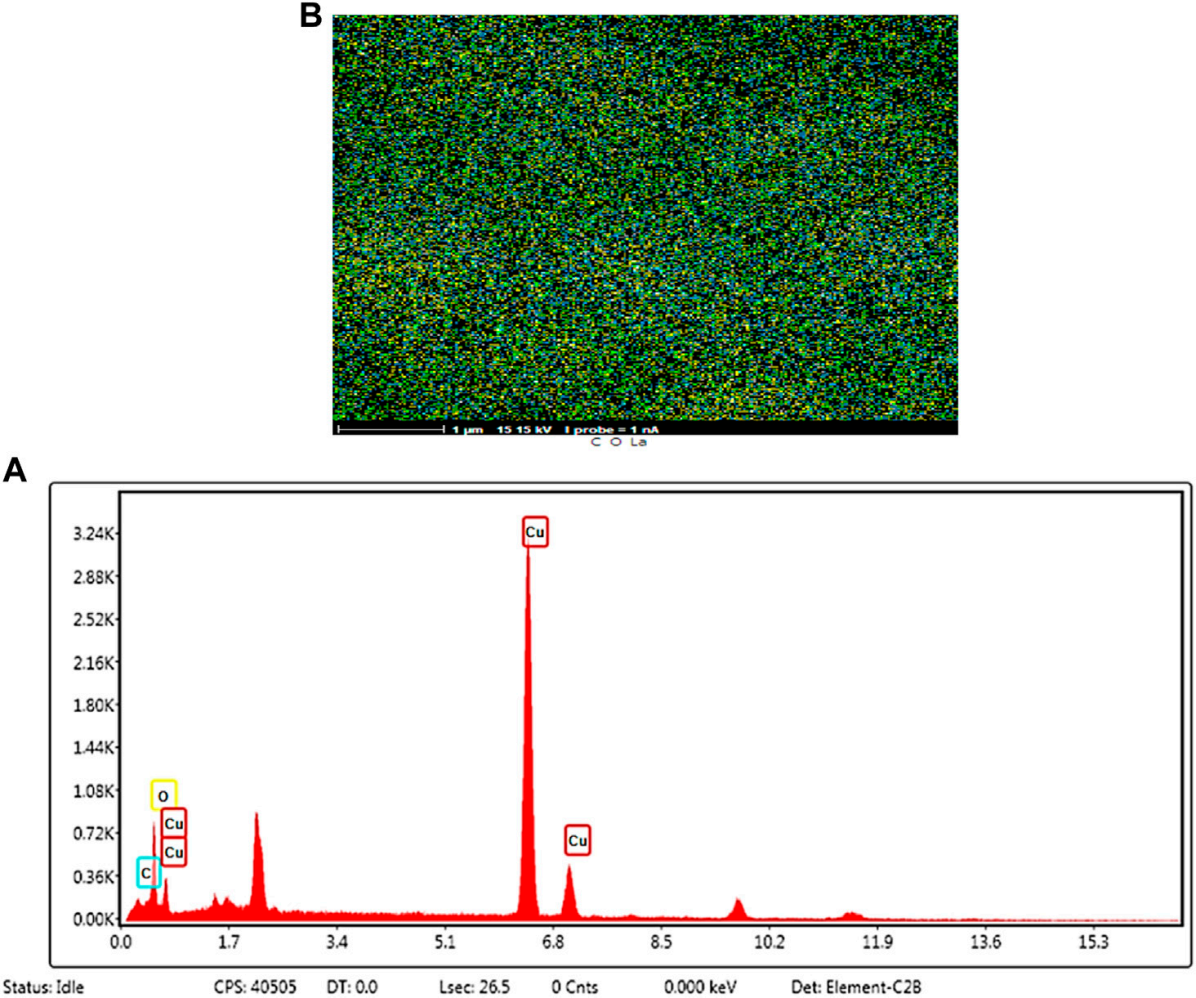
EDS elemental analysis **(A)** and mapping graph **(B)** of Cu/DPA-MOF/OP/CS hydrogel.

Thermal stability is one of the critical factors in the application of nanocompounds and polymers. The TGA curve of Cu/DPA-MOF (A) and Cu/DPA-MOF/OP/CS hydrogel (B), which shows their thermal stability, is presented in Figure 5. In both samples, the first weight loss is related to water loss on the surface and water enclosed in the structure, which is visible in the region around 90°C–110°C. In Cu/DPA-MOF, the observed weight loss from temperatures above 200°C to areas below 400°C can be attributed to the decomposition of main structures. Based on the thermal behavior of the Cu/DPA-MOF/OP/CS hydrogel, in the region (200°C–400°C), the structures of chitosan and oxidized pectin may have disappeared. As an important result, the Cu/DPA-MOF/OP/CS hydrogel has more thermal stability than Cu/DPA-MOF. This behavior can be attributed to the hydrogel nature of the final structures. Therefore, the Cu/DPA-MOF/OP/CS hydrogel is completely stable up to a higher temperature, and this feature can be used in various applications (Figure 6).

In order to ensure the formation of Cu/DPA-MOF/OP/CS hydrogel products, EDS elemental analysis with the mapping graph has been carried out. It is observed that the amounts of elements of carbon, oxygen, and Cu are shown in EDS analysis. In addition, the presence of these elements has been confirmed schematically in Fig 6. As an important result, the presence of these elements in the final structures is a strong evidence for the formation of the Cu/DPA-MOF/OP/CS hydrogel structure.

**FIGURE 7:**
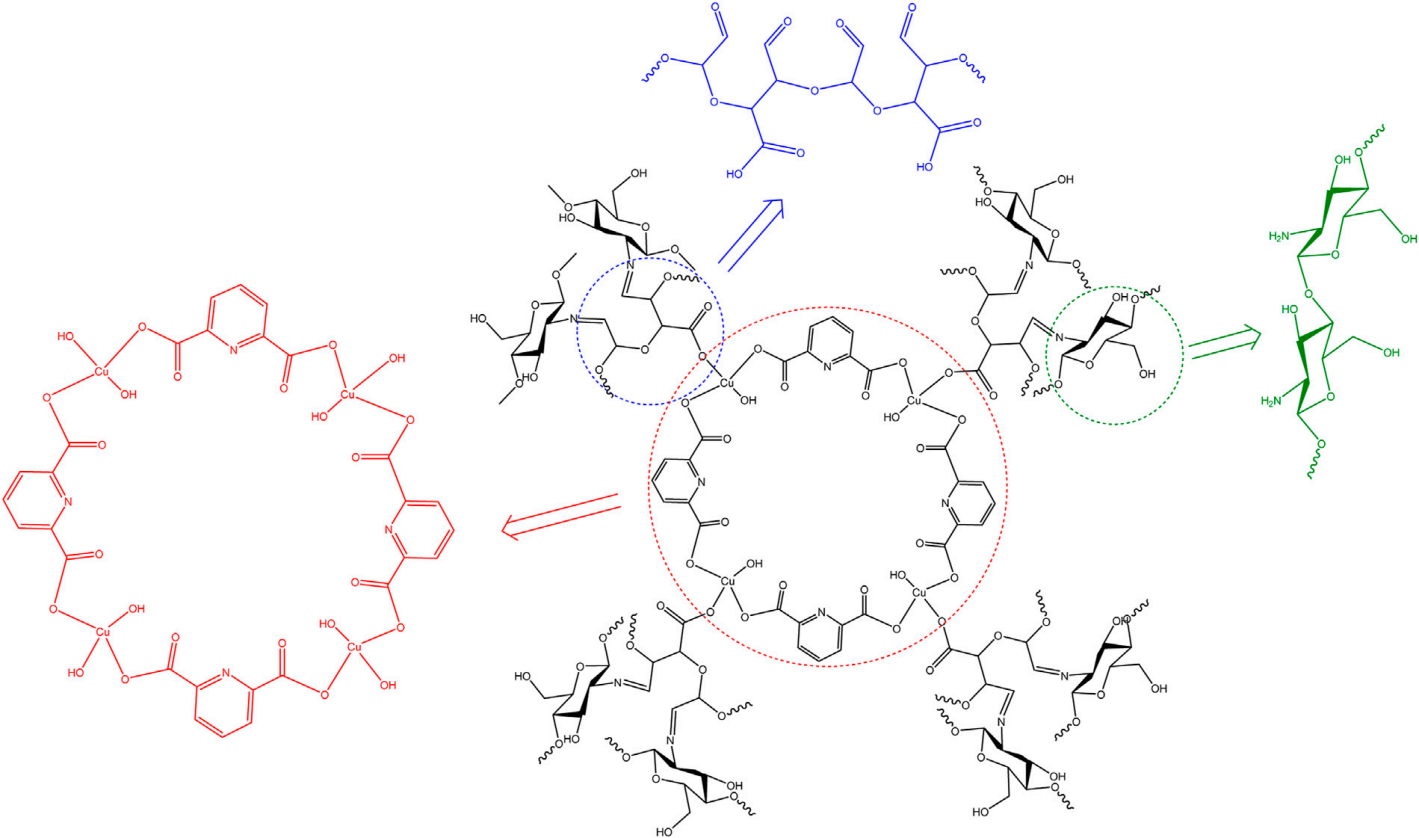
Proposed structure of Cu/DPA-MOF/OP/CS hydrogel.

Using the interpreted analysis, especially FT-IR spectra, XRD patterns, and EDS elemental analysis with the mapping graph, it is suggested that the Cu/DPA-MOF/OP/CS hydrogel was synthesized in the form, as shown in Figure 7.

As shown in Figure 7, the proposed structure is consistent with the general groups observed during FT-IR spectra.

### 3.2 Antimicrobial effects of Cu/DPA-MOF and the Cu/DPA-MOF/OP/CS hydrogel

In the antimicrobial evaluations of Cu/DPA-MOF and the Cu/DPA-MOF/OP/CS hydrogel, cefazolin, as a commercial antibacterial drug, and terbinafine, as a commercial antifungal drug, against five Gram-negative species, five Gram-positive species, and three fungal species were tested and investigated. The results of antimicrobial activities are given in Tables 1, 2 and Table 3.

**TABLE 1:** Antimicrobial effects of Cu/DPA-MOF and Cu/DPA-MOF/OP/CS hydrogel on Gram-negative species.

Compound GN-species		Cu/DPA-MOF	Cu/DPA-MOF/OP/CS hydrogel	Cefazolin
*Klebsiella pneumoniae* (ATCC 13883)	MIC	4	4	1
	MBC	8	8	2
	DIZD	17.04	18.26	17.53
*Escherichia coli* (ATCC 25922)	MIC	64	32	32
	MBC	128	64	64
	DIZD	16.61	19.82	22.37
*Yersinia enterocolitica* (ATCC 9610)	MIC	32	16	16
	MBC	64	32	32
	DIZD	17.46	17.53	16.16
*Proteus mirabilis* (ATCC 7002)	MIC	32	16	32
	MBC	64	32	64
	DIZD	16.43	15.29	18.61
*Acinetobacter baumannii* (ATCC 19606)	MIC	64	32	—
	MBC	128	64	—
	DIZD	14.28	16.97	—

GN-species: Gram-negative species

DIZD, reported as mm

MIC, reported as μg/mL

MBC, reported as μg/mL

**TABLE 2:** Antimicrobial effects of Cu/DPA-MOF and Cu/DPA-MOF/OP/CS hydrogel on Gram-positive species.

Compound GP-species		Cu/DPA-MOF	Cu/DPA-MOF/OP/CS hydrogel	Cefazolin
*Listeria monocytogenes* (ATCC 19115)	MIC	64	64	16
	MBC	128	32	32
	DIZD	20.37	22.16	21.73
*Staphylococcus epidermidis* (ATCC 14990)	MIC	8	4	4
	MBC	16	8	8
	DIZD	17.95	17.42	18.59
*Bacillus cereus* (ATCC 11778)	MIC	64	64	—
	MBC	128	128	—
	DIZD	14.36	15.67	—
*Rhodococcus equi* (ATCC 25729)	MIC	128	32	—
	MBC	256	64	—
	DIZD	14.34	16.18	—
*Staphylococcus aureus* (ATCC 29213)	MIC	2	1	2
	MBC	4	2	4
	DIZD	18.43	17.97	21.21

GP-species: Gram-positive species

DIZD, reported as mm

MIC, reported as μg/mL

MBC, reported as μg/mL

**TABLE 3:** Antimicrobial effects of Cu/DPA-MOF and Cu/DPA-MOF/OP/CS hydrogel on fungal species.

Compound		Cu/DPA-MOF	Cu/DPA-MOF/OP/CS hydrogel	Terbinafine
F species				
*Candida albicans* (ATCC 10231)	MIC	32	8	64
	MFC	64	16	128
	DIZD	18.64	20.14	16.38
*Fusarium oxysporum* (ATCC 7601)	MIC	32	16	32
	MFC	64	32	64
	DIZD	18.37	20.53	21.15
*Aspergillus fumigatus* (ATCC 1022)	MIC	16	16	16
	MFC	32	32	32
	DIZD	18.62	19.31	19.96

F species: fungal species

DIZD, reported as mm

MIC, reported as μg/mL

MFC, reported as μg/mL

During the investigation of antibacterial and antimicrobial activities, it is proved from the results reported in the tables that the effectiveness of Cu/DPA-MOF and Cu/DPA-MOF/OP/CS hydrogel was more than cefazolin and terbinafine, which are known drugs. As is known, copper is a disinfectant compound with significant antimicrobial properties. The observed powerful antimicrobial properties of the synthesized compounds can be attributed to the copper complex present in the structure, the nano-sized structures, and the high specific surface area.

Comparing the effects of Cu/DPA-MOF and Cu/DPA-MOF/OP/CS hydrogel shows that the Cu/DPA-MOF/OP/CS hydrogel was relatively more effective than Cu/DPA-MOF. The reason can be attributed to the specific surface area, and as mentioned, a higher specific surface area causes more effectiveness and efficiency in nanoparticles (Asiri et al., 2022; Suksatan et al., 2022; Zeraati et al., 2022).

## 4 Conclusion

Briefly, in this study, the novel Cu/DPA-MOF/OP/CS hydrogel was synthesized in mild conditions with remarkable physichochemical properties. To identify and confirm the structure of the novel hydrogel synthesized, techniques such as N_2_ adsorption/desorption isotherm, FT-IR spectrum, XRD patterns, SEM images, thermal stability curves, EDS elemental analysis, and mapping graph were used. Particle size in the nano range, high specific surface area, and high thermal stability were the essential features of the synthesized hydrogel. Next, the antimicrobial properties (antibacterial and antifungal) of the synthesized compounds were tested. To evaluate the antimicrobial properties of the synthesized compounds, the antimicrobial properties of cefazolin and terbinafine, which are well-known drugs in the market, were assessed and compared with the effects of synthetic nanoparticles. A comparison of the results showed that the synthesized compounds had higher antimicrobial properties than drugs. The observed significant antimicrobial properties of the synthesized compounds can be attributed to the efficient network complex present in the structure, the nano-sized structures, and the high specific surface area with stable porosity.

## Data Availability

The original contributions presented in the study are included in the article/Supplementary Material; further inquiries can be directed to the corresponding author.
